# Phospholipase A/Acyltransferase enzyme activity of H-rev107 inhibits the H-RAS signaling pathway

**DOI:** 10.1186/1423-0127-21-36

**Published:** 2014-05-01

**Authors:** Chun-Hua Wang, Rong-Yaun Shyu, Chang-Chieh Wu, Tzung-Chieh Tsai, Lu-Kai Wang, Mao-Liang Chen, Shun-Yuan Jiang, Fu-Ming Tsai

**Affiliations:** 1 Department of Dermatology, Taipei Tzuchi Hospital, The Buddhist Tzuchi Medical Foundation, New Taipei City, Taiwan; 2 Department of Internal Medicine, Taipei Tzuchi Hospital, The Buddhist Tzuchi Medical Foundation, New Taipei City, Taiwan; 3 School of Medicine, Tzu Chi University, Hualien, Taiwan; 4 Department of Surgery, Tri-Service General Hospital, National Defense Medical Center, Taipei, Taiwan; 5 Department of Microbiology, Immunology and Biopharmaceuticals, National Chiayi University, Chiayi, Taiwan; 6 Graduate Institute of Life Sciences, National Defense Medical Center, Taipei, Taiwan; 7 Department of Research, Taipei Tzuchi Hospital, The Buddhist Tzuchi Medical Foundation, New Taipei City, Taiwan

**Keywords:** H-rev107, HRASLS3, PLA2G16, H-RAS, Phospholipase A/acyltransferase, Acyl-biotin exchange assay

## Abstract

**Background:**

H-rev107, also called HRASLS3 or PLA2G16, is a member of the HREV107 type II tumor suppressor gene family. Previous studies showed that H-rev107 exhibits phospholipase A/acyltransferase (PLA/AT) activity and downregulates H-RAS expression. However, the mode of action and the site of inhibition of H-RAS by H-rev107 are still unknown.

**Results:**

Our results indicate that H-rev107 was co-precipitated with H-RAS and downregulated the levels of activated RAS (RAS-GTP) and ELK1-mediated transactivation in epidermal growth factor-stimulated and H-RAS-cotransfected HtTA cervical cancer cells. Furthermore, an acyl-biotin exchange assay demonstrated that H-rev107 reduced H-RAS palmitoylation. H-rev107 has been shown to be a PLA/AT that is involved in phospholipid metabolism. Treating cells with the PLA/AT inhibitor arachidonyl trifluoromethyl ketone (AACOCF3) or methyl arachidonyl fluorophosphate (MAFP) alleviated H-rev107-induced downregulation of the levels of acylated H-RAS. AACOCF3 and MAFP also increased activated RAS and ELK1-mediated transactivation in H-rev107-expressing HtTA cells following their treatment with epidermal growth factor. In contrast, treating cells with the acyl-protein thioesterase inhibitor palmostatin B enhanced H-rev107-mediated downregulation of acylated H-RAS in H-rev107-expressing cells. Palmostatin B had no effect on H-rev107-induced suppression of RAS-GTP levels or ELK1-mediated transactivation. These results suggest that H-rev107 decreases H-RAS activity through its PLA/AT activity to modulate H-RAS acylation.

**Conclusions:**

We made the novel observation that H-rev107 decrease in the steady state levels of H-RAS palmitoylation through the phospholipase A/acyltransferase activity. H-rev107 is likely to suppress activation of the RAS signaling pathway by reducing the levels of palmitoylated H-RAS, which decreases the levels of GTP-bound H-RAS and also the activation of downstream molecules. Our study further suggests that the PLA/AT activity of H-rev107 may play an important role in H-rev107-mediated RAS suppression.

## Background

H-rev107 [1], also called HRASLS3 [2] or PLA2G16 [3], was first identified in H-RAS-resistant murine fibroblasts [4]. H-rev107 is a member of the HREV107 type II tumor suppressor gene family, which includes H-REV107, retinoid-inducible gene 1 (RIG1) [5], HRASLS2 [6,7], HRLP5 [8], and HRASLS (A-C1) [9,10], the last of which is present in humans, rats, and mice. In this family, the protein contains an NC domain at the N-terminus and a hydrophobic membrane-anchoring domain at the C-terminus [11,12]. The proteins in this family exhibit activities that regulate cellular growth, differentiation, and apoptosis [13-20].

Some research results provide information about the molecular mechanisms underlying the biological functions of HREV107 family proteins. Murine H-rev107, human H-REV107, and human HRASLS were also shown to inhibit RAS-mediated transformation of fibroblasts, EC cells, and tumor cell lines [4,9,21]. Similar inhibition of RAS signaling pathways has been observed in HRASLS2-expressing [7] or RIG1-expressing cervical and gastric cancer cells [13,19,20]. In keratinocytes, RIG1 has been shown to stimulate cellular differentiation that is mediated by activating type I tissue transglutaminase or regulating tubulin to drive the formation of the peripheral microtubule ring [15,17,18,22]. HREV107 family proteins also exhibit proapoptotic activities. The proapoptotic activities of RIG1 are mediated through caspase-dependent [14,18,19] or -independent pathways [14,15] in normal keratinocytes and cancer cells. H-REV107 has been shown to be a target of interferon-regulatory factor-1 and to be involved in IFN-γ-induced cell death in human ovarian carcinoma cells [23].

HREV107 family proteins have recently been considered phospholipid-metabolizing enzymes. The release of free fatty acids and lysophospholipid from phosphatidylcholine catalyzed by H-REV107 indicates that H-REV107 acts as a phospholipase A [3,24]. Furthermore, phospholipid-related enzyme activities have also been identified for RIG1, HRASLS2, A-C1, and HRLP5 [6,8,25]. All members of this family possess phospholipase A_1/2_ (PLA_1/2_) activity as well as O-acyltransferase activity (i.e., PLA/AT). Although similar enzyme activities were observed, each member exhibits a different spectrum of PLA/AT activity. HRASLS2 exhibits higher PLA_1/2_ activity, whereas O-acylation activity has been observed in H-REV107 and RIG1 [6].

RAS proteins are plasma membrane associated GTPases. Different isoforms of RAS (H-, N-, and K-RAS) can regulate a variety of cellular processes involving proliferation, differentiation, and apoptosis. RAS activity is regulated by guanidine nucleotide exchange factors (GEFs) and GTPase-activating proteins (GAPs). GEFs activate RAS by releasing GDP and permitting GTP binding, whereas GAPs inactivate RAS by catalyzing the hydrolysis of bound GTP to return RAS to the GDP-bound state [26-28]. In addition to GTP binding, RAS proteins must associate with the plasma membrane to transduce their signals. The CAAX motif of RAS directs the post-translational modification of the C-terminus of the RAS protein with a polyisoprenoid lipid [29,30]. This lipid modification is required for the trafficking of RAS from the endomembrane system to the plasma membrane [31]. Palmitoylation (S-acylation) is readily reversible, and the cycle of depalmitoylation and repalmitoylation is linked to RAS trafficking to and from the Golgi apparatus [32-34].

The entire HREV107 protein family can inhibit the RAS signaling pathway. The exact molecular mechanism by which H-rev107 inhibits RAS is still unknown. H-rev107 has been shown to possess PLA/AT activity, and the activation of H- or N-RAS is tightly regulated by its cellular localization with modification by de- and reacylation. We hypothesized that H-rev107 may act as a PLA/AT enzyme to regulate RAS lipid modification and subsequently affect RAS activity. To discern the role of H-rev107 on RAS palmitoylation, we determined the acylated status of H-RAS in cells expressing H-rev107. Here we show that H-rev107 can decrease the level of acylated H-RAS. We also show that H-rev107, via its PLA/AT activity, inhibits the RAS signaling pathway.

## Methods

### Expression vectors and materials

The vectors pH-rev107-myc and pH-RAS have been described previously [13,35]. The vectors pH-rev107-his and pH-rev107 (1–132)-his, which encode, respectively, full-length H-rev107 and H-rev107 with 30 amino acids truncated at the C-terminus (1–132), were constructed by amplification from pH-rev107-myc plasmid using primers for pH-rev107 full length (FL) (5’- CGGGATCCATGCTAGCACCCATACC-3’ and 5’-GCAAGCTTTTGCTTCTGTTTCTTGTTTCTGGAGAG-3’) and pH-rev107 1–132 (5’-CGGGATCCATGCTAGCACCCATACC-3’ and 5’- GCAAGCTTATCTCTGACCTGATCACTCCG-3’). The amplified cDNA fragments were then subcloned inframe into the *Bam*HI-*Hind*III sites of the pET-29a (+) vectors (Novagen, EMD Bioscience, MA, USA). The cDNA sequences of the fusion proteins were confirmed by DNA sequencing. Arachidonyl trifluoromethyl ketone (AACOCF3) was purchased from Cayman Chemical (Ann Arbor, MI, USA). Palmostatin B was purchased from Millipore Corporation (Billerica, MA, USA). Bromoenol lactone (BEL) and methyl arachidonyl fluorophosphate (MAFP) were purchased from Sigma (St. Louis, MO, USA).

### Protein expression and purification

Both full length and C-terminal truncated H-rev107 were isolated and purified from *E. coli* over-expressing the respective proteins. Expression of the inserted gene is via the induction of T7 polymerase with 1 mM isopropyl β-D-1-thiogalactopyranoside in BL21(DE3) *E. coli* after cells reach mid-log phase. Purification of the protein was facilitated by the polyhistidine tag at the C-terminus of the H-rev107 fusion proteins. Purified proteins were confirmed by Western blot analysis using antibodies against polyhistidine (Sigma) (data not shown).

### Cell culture and transfection

HtTA cervical cancer cells [20] were maintained in RPMI-1640 medium supplemented with 25 mM HEPES, 26 mM NaHCO_3_, 2 mM L-glutamine, penicillin (100 units/mL), streptomycin (100 μg/mL), and 10% fetal bovine serum (FBS) at 37°C in an atmosphere of 5% CO_2_ in air. For DNA transfection, cells plated in culture dishes were transfected with the expression vectors using the method of liposome-mediated transfection. Briefly, plasmids and lipofectamine 2000 (Gibco BRL, Gaithersburg, MD, USA) were separately diluted in Opti-MEM medium and then mixed together and incubated at room temperature for 15 min. The DNA-lipofectamine complexes were then incubated with the cells for 4 h at 37°C. Cells were refreshed with complete medium for 24 h and then incubated in medium without serum for 12 h. Cells were then stimulated with epidermal growth factor (EGF, 50 ng/mL; Sigma) for 5 min at 37°C before harvesting. For protein transfection, cells plated in culture dishes were transfected with H-rev107(FL) or H-rev107(1–132) using a protein delivery reagent (PULsin®, Polyplus, BP, France). Protein was diluted in 20 mM Hepes buffer (pH 7.4) and then mixed with PULsin® reagent at room temperature for 15 min. The protein-PULsin® reagent complexes were then incubated with the cells for 4 h at 37°C.

### RAS activity assay

RAS activity was assessed using the RAS activation assay kit (Upstate Biotechnology, Lake Placid, NY, USA). Briefly, cells in a 10-cm dish were washed twice with ice-cold phosphate buffered saline (PBS) and then lysed in 0.5 mL of MLB buffer (25 mM HEPES, pH 7.5, 150 mM NaCl, 1% Igepal CA-630, 10 mM MgCl_2_, 1 mM EDTA, and 10% glycerol) containing 1× complete protease inhibitor cocktail (EDTA-free) (Roche Diagnostics, Mannheim, Germany) and phosphatase inhibitors (2 mM NaF and 1 mM Na_3_VO_4_). After centrifugation at 14,000 × g for 5 min, protein in the lysate was quantified using the Bio-Rad protein assay kit (Bio-Rad Laboratories, Hercules, CA, USA). Cellular lysates containing 300 μg of protein were then incubated at 4°C for 45 min with 5 μL of RAF-1 RBD agarose bound with glutathione S-transferase fusion protein corresponding to the human RAS binding domain (RBD, residues 1–149) of Raf-1. After washing three times with MLB that contains protease and phosphatase inhibitors, the presence of the activated RAS (RAS-GTP) was detected by Western blotting using an anti-RAS monoclonal antibody that recognizes RAS (Clone 10, Upstate Biotechnology).

### ELK1 reporter assay

The ELK1 pathway was used to analyze the effects of H-rev107 on H-RAS induced by EGF using the PathDetect® in vivo signal transduction pathway transreporting system (Stratagene, La Jolla, CA, USA). The cells were plated overnight in triplicate in six-well plates at a density of 2 × 10^5^ cells per well in RPMI-1640 medium that contains 10% FBS. The cells were then cotransfected with an H-rev107 expression vector or empty control vector, H-RAS expression vector, the transactivator plasmid pFA-ELK1, and the reporter plasmid pFR-luc. After transfection, the cells were incubated for 24 h in medium that contains 1% FBS and then cultured for 24 h in serum-free medium containing 10 μM AACOCF3 and either 1 μM palmostatin B or dimethyl sulfoxide (DMSO) vehicle (0.1%). To analyze the activity of the transfected HRAS, the cells were stimulated with EGF (50 ng/mL) for 12 h at 37°C before harvesting. Luciferase activity was then measured using the luciferase assay kit (Stratagene) and a multi-functional microplate reader (Infinite F200, Tecan, Durham, NC, USA). The relative luciferase activity of each sample was determined after normalizing the protein concentration of each lysate. All experiments were performed in triplicate.

### Immunoprecipitation and Western blotting

Cells were lysed in MLB that contains protease and phosphatase inhibitors. Cell lysates containing 500 μg of protein were first incubated for 2 h at 4°C with 3.2 μg of anti-MYC (Invitrogen, Carlsbad, CA, USA) or anti-RAS (Upstate Biotechnology) monoclonal antibody and then incubated for 2 h at 4°C with 20 μL of protein G plus/protein A agarose (Calbiochem, Cambridge, MA, USA). Immunoprecipitated complexes were analyzed by Western blotting using an anti-RAS or anti-MYC antibody after washing three times with PBS. For Western blotting, 20 μg of protein were separated on 15% polyacrylamide gels and transferred to polyvinylidene fluoride membranes. After blocking, membranes were incubated for 12 h at 4°C with anti-MYC, anti-RAS, anti-polyhistidine, or anti-actin (Sigma) antibody and then incubated with horseradish peroxidase-conjugated goat anti-mouse antibody at room temperature for 1 h. An ECL kit (Amersham, Bucks, UK) was used to detect the substrate reaction. The relative protein expression was quantified following normalization with the levels of the actin.

### Acyl-biotin exchange assay

Cells plated in 10-cm dishes were transfected with 5 μg of expression plasmids and then incubated for 24 h in complete medium containing 10 μM AACOCF3 and either 1 μM palmostatin B or DMSO vehicle (0.1%). Cells were lysed in 1 mL of lysis buffer (50 mM Tris–HCl, pH 7.2, 1% Triton X-100, 150 mM NaCl, 1% sodium deoxycholate, 1 mM EDTA, 0.1% SDS, and protease and phosphatase inhibitors containing 10 mM N-ethylmaleimide) (Sigma). Acyl-biotin exchange assays were performed as described previously with minor modifications [36]. Briefly, 5 μg of protein were precipitated using chloroform-methanol and treated exhaustively with N-ethylmaleimide to block free thiol groups, which was removed by sequential chloroform-methanol precipitation. The samples were then treated with hydroxylamine (Sigma) and EZ-Link® HPDP-Biotin (Pierce Biotechnology, Rockford, IL, USA) to exchange thiol-bound fatty acids for biotin. Equal amounts of solubilized protein were incubated with 15 μL of streptavidin agarose resin (Pierce Biotechnology) at 25°C for 90 min. Immunoprecipitated complexes were analyzed by Western blotting using an anti-RAS antibody after washing three times with washing buffer (50 mM Tris–HCl, pH 7.4, 150 mM NaCl, 5 mM EDTA, 0.2% Triton X-100, and 0.1% SDS).

## Results

### H-rev107 inhibited H-RAS activation induced by EGF

**Figure 1:**
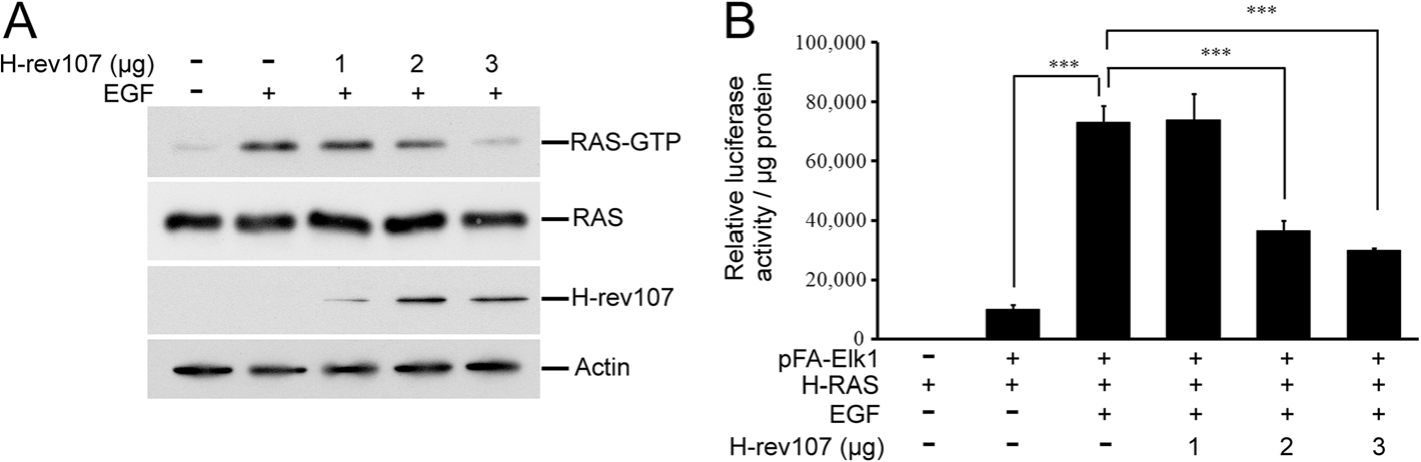
**H-rev107 inhibited activation of H-RAS induced by EGF. (A)** HtTA cells plated in 10-cm dishes were transfected with H-rev107 or control vector for 24 h and then serum starved for 12 h. Cells were stimulated with EGF (50 ng/mL) for 5 min. Cell lysates were prepared, and the activated RAS (RAS-GTP) was isolated with RAF-RBD-conjugated agarose. The levels of total RAS, H-rev107 fusion proteins, and actin were measured by Western blotting. **(B)** HtTA cells were plated in triplicate, incubated overnight, transfected for 2 days with control vector, H-rev107 expression vector, and reporter plasmids, and were then stimulated with EGF (50 ng/mL) for 12 h at 37°C. Cell lysates were prepared and analyzed for the transactivation activity of ELK1 protein. Representative results from triplicate samples are expressed as mean ± SD. Student’s *t*-test: ***, *P* < 0.001.

Since H-rev107 was first isolated from revertants of H-RAS-transformed fibroblasts, we examined the effect of H-rev107 on the activation of H-RAS. Stimulation of HtTA cells with EGF significantly enhanced activated RAS (RAS-GTP) by about 12.3-fold (Figure 1A). The EGF-stimulated RAS-GTP levels were suppressed in a dose-dependent manner with maximal 86.6% inhibition in cells transfected with 3 μg of H-rev107 expression vector (Figure 1A).

We next examined the effects of H-rev107 on ELK1 transactivation in cells stimulated with EGF for 12 h. ELK1 transactivation dramatically increased by 7.4-fold in EGF-treated HtTA cells. We also observed a dose-dependent downregulation of ELK1 transactivation in H-rev107-expressing HtTA cells (Figure 1B).

### H-rev107 was associated with H-RAS and inhibited its palmitoylation

**Figure 2:**
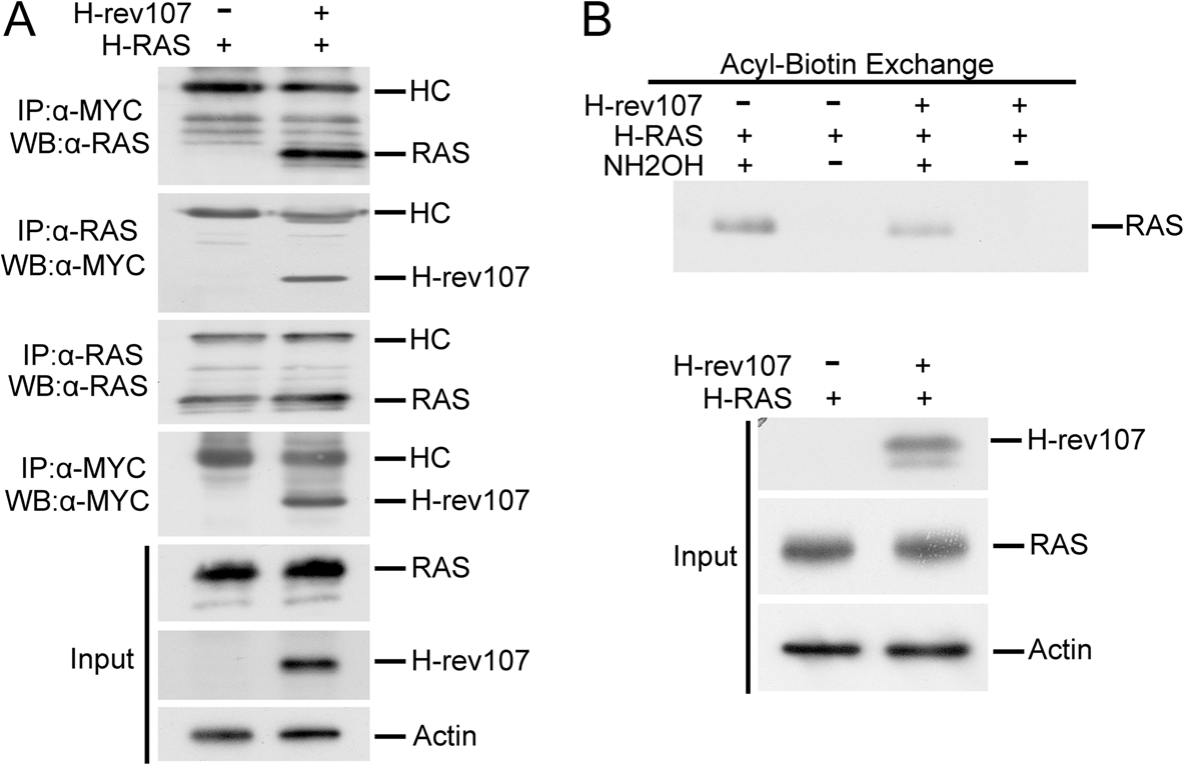
**H-rev107 was associated with H-RAS and inhibited its palmitoylation. (A)** HtTA cells plated in a 10-cm dish were transfected for 24 h with 0.1 μg of H-RAS along with H-rev107-myc or the control vector. Cell lysates were prepared as described in Methods. The interaction between H-rev107 and RAS was analyzed by immunoprecipitation followed by Western blot analysis. **(B)** HtTA cells plated in a 10-cm dish were transfected for 24 h with 0.1 μg of H-RAS along with 3 μg of H-rev107-myc or the control vector. Cell lysates were prepared, and acyl-biotin exchange analysis of H-RAS was performed as described in Methods. Aliquots containing 5 μg of protein including acylated RAS were biotinylated and then processed with streptavidin agarose resin followed by Western blot analysis. The input consisted of 300 ng of protein from the acyl-biotin exchange that was loaded. HC: heavy chain; NH_2_OH: hydroxylamine.

The results above suggest that H-rev107 can inhibit H-RAS activation. We then analyzed whether H-RAS is the acylated target of H-rev107. We first performed co-immunoprecipitation with lysates of HtTA cells that coexpressed H-RAS along with empty vector or H-rev107-myc fusion protein. Our analysis revealed that H-RAS was immunoprecipitated along with H-rev107 fusion protein using anti-MYC antibody against the MYC epitope of the H-rev107 fusion protein. Similarly, H-rev107 was detected in the H-RAS immunoprecipitate (Figure 2A).

Palmitoylation or S-acylation is the post-translational attachment of fatty acids to cysteine residues of RAS. This modification is essential for the specific subcellular distribution and the GTP-GDP exchange of RAS [29,30]. H-rev107 has been reported to act as a PLA/AT and to play a role in the regulation of adipocyte metabolic processes [3,37]. We then used an acyl-biotin exchange assay to examine the effect of H-rev107 on H-RAS palmitoylation. As shown in Figure 2B, the total amount of H-RAS in the lysates of H-rev107-expressing cells did not differ from that in control cell lysates. However, the amount of H-RAS captured on the biotin-labeled HPDP was decreased by 50.5% in H-rev107-transfected lysates treated with hydroxylamine. No acylated H-RAS protein was detected in the cell lysate in the absence of hydroxylamine, demonstrating the presence of hydroxylamine-sensitive linkages. This result suggests that H-rev107 decreases the levels of H-RAS by modifying lipid acylation of H-RAS.

**Figure 3:**
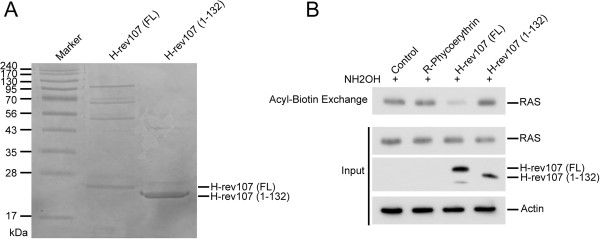
**H-rev107 protein inhibited RAS palmitoylation. (A)** Coomassie blue staining of purified full-length and C-terminal-truncated H-rev107 from *E. coli*. The 6 × His-tagged fusion proteins of H-rev107 were purified using a HiTrap™ Chelating HP column. The purified proteins were subjected to 12% SDS-PAGE. **(B)** HtTA cells plated in a 6-cm dish were transfected for 24 h with 7 μg of H-rev107 (FL) or R-phycoerythrin or 3 μg of H-rev107 (1–132) protein. Cell lysates were prepared, and acyl-biotin exchange analysis of H-RAS was performed as described in Methods. Acylated RAS from 5 μg of cytosolic extract was collected with streptavidin agarose resin followed by analysis by Western blotting. The loading input consisted of 300 ng of protein from the acyl-biotin exchange. FL: full length.

We next determined the effect of purified H-rev107 protein on RAS palmitoylation. Polyhistidine-tagged H-rev107 (FL) or H-rev107 (1–132) fusion proteins with the expected molecular weights of 22.9 and 20 kDa, respectively, were detected in cytosolic extracts prepared and purified from BL21 (DE3) *E. coli* cells following induction by incubation with isopropyl β-D-1-thiogalactopyranoside for 4 h (Figure 3A). The purity of the full-length and C-terminal truncated H-rev107 proteins reached 38.2% and 84.7%, respectively. Similar to results observed in Figure 2B, the levels of acylated RAS were decreased by 74.7% in HtTA cells transfected with purified H-rev107 (FL) protein compared to the cells transfected with the control protein R-phycoerythrin (Figure 3B). Although a higher level of purity was attained for the C-terminal truncated H-rev107 protein [H-rev107(1–132)], this protein had no effect on the levels of acyl-RAS in HtTA cells.

### PLA/AT inhibitor eliminated H-rev107-mediated inhibition of H-RAS palmitoylation

**Figure 4:**
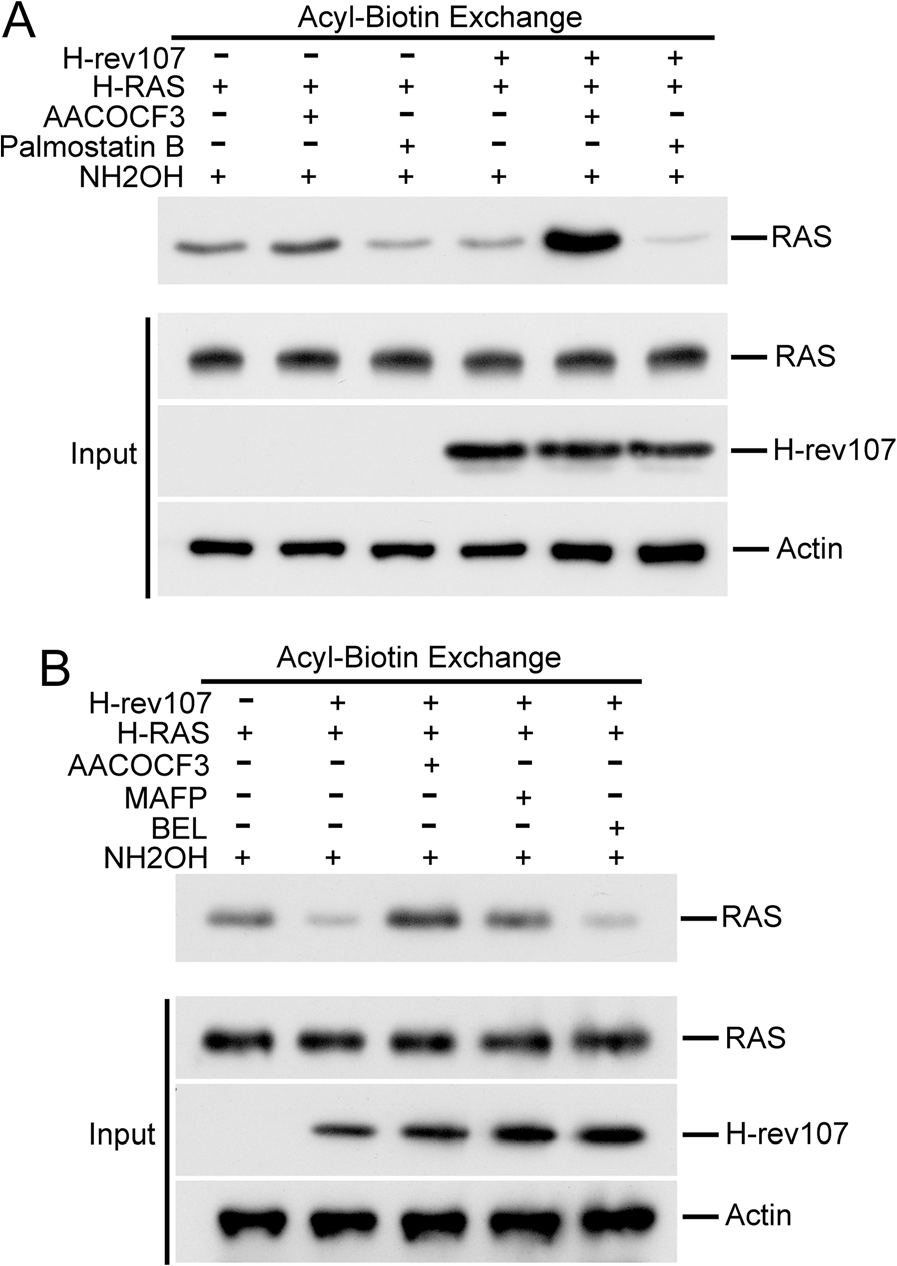
**AACOCF3 and MAFP eliminated H-rev107-mediated H-RAS palmitoylation.** HtTA cells plated in a 10-cm dish were transfected for 24 h with 0.1 μg of H-RAS along with H-rev107-myc or the control vector in the presence of 10 μM AACOCF3 **(A, B)**, 1 μM palmostatin B **(A)**, 5 μM MAFP **(B)**, 2 μM BEL **(B)** or DMSO vehicle. Cell lysates were prepared, and acyl-biotin exchange analysis of H-RAS was performed as described in Methods. Levels of H-RAS and H-rev107 in the cell lysates are shown in the bottom panel. NH_2_OH: hydroxylamine.

Previous studies demonstrated that cycles of de- and re-acylation can regulate the activity of H-RAS [32,34]. Protein palmitoylation is carried out by palmitoyl acyltransferases (PATs) during deacylation by acyl-protein thioesterases (APTs) [32,38]. We next determined whether H-rev107 affected H-RAS palmitoylation via acyl-protein thioesterases (APT1) or PLA/AT-induced deacylation/acylation. We did this analysis by using, respectively, an APT1 inhibitor (palmostatin B) [39] or a PLA/AT inhibitor (AACOCF3) [3]. We observed that treating HtTA cells with AACOCF3 slightly increased acylated H-RAS by 31%. However, acylation of H-RAS was decreased by 47.6% when cells were treated with palmostatin B in control transfected cells. Treating H-rev107-expressing HtTA cells with AACOCF3 increased acylated H-RAS by 4-fold (Figure 4A), whereas treatment with palmostatin B decreased the levels of acylated H-RAS by 82.7%.

In order to elucidate the effect of PLA/AT activity on H-rev107-mediated H-RAS acylation, we performed a series of studies with commercially available PLA2 inhibitors. AACOCF3 and MAFP have been shown to inhibit the activity of cytosolic human phospholipase A2, whereas BEL is a mechanism-based suicide substrate inhibitor of calcium-independent PLA2. Also, BEL has been shown to inhibit lipases that contain the GXSXG consensus motif [40-43]. Expression of H-rev107 resulted in downregulation of acylated H-RAS by 75.6%. AACOCF3 and MAFP increased acylated H-RAS by 6.4- and 3.8-fold, respectively, in H-rev107-expressing HtTA cells (Figure 4B). BEL had no effect on acylated H-RAS production in H-rev107-expressing HtTA cells.

### PLA/AT inhibitor eliminated H-rev107-mediated inhibition of H-RAS activation induced by EGF

**Figure 5:**
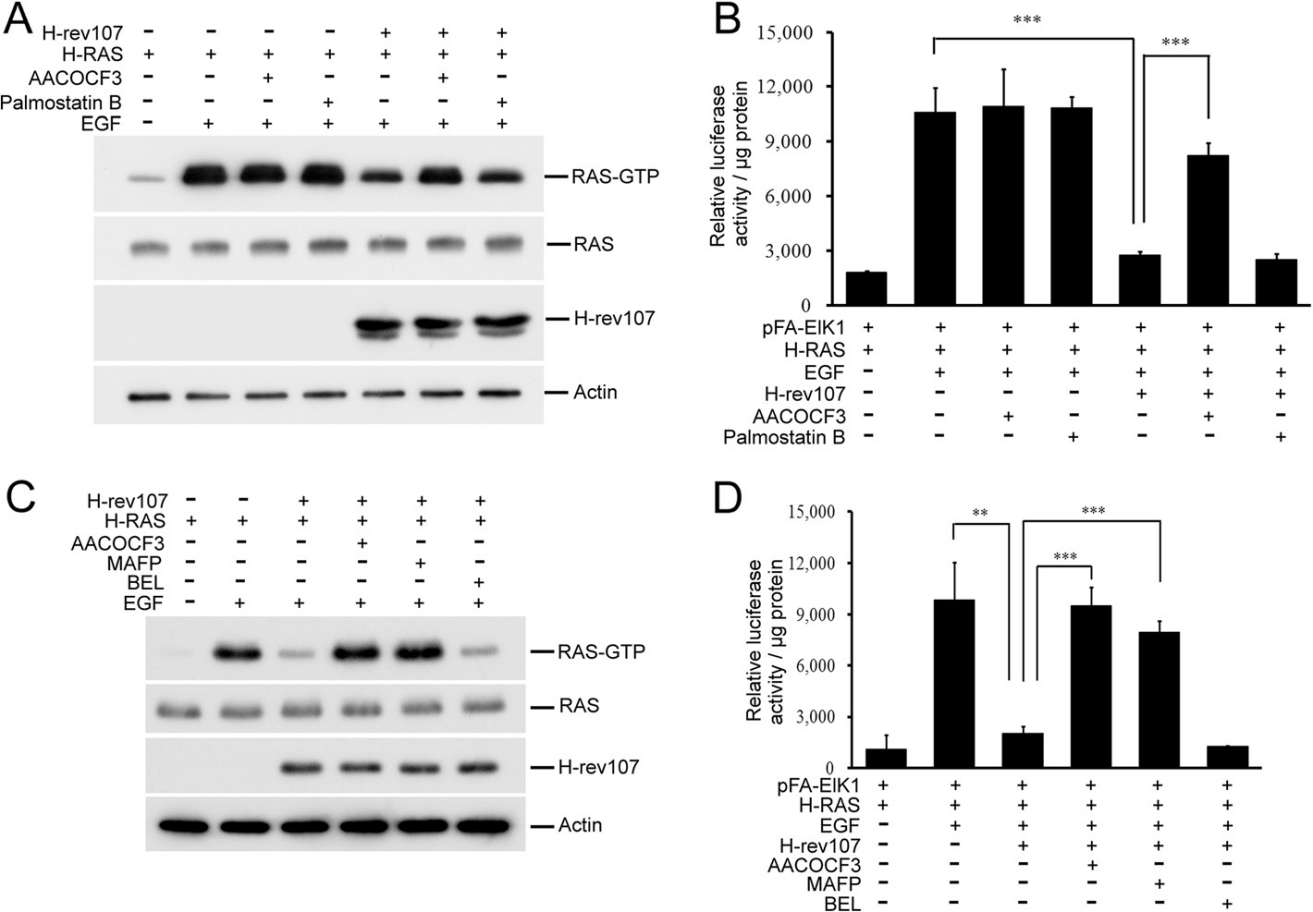
**AACOCF3 and MAFP eliminated H-rev107 inhibition of H-RAS activation induced by EGF. (A, C)** HtTA cells plated in 10-cm dishes were transfected for 24 h with H-rev107 or control vector in the presence of 10 μM AACOCF3, 5 μM MAFP, 2 μM BEL, 1 μM palmostatin B or DMSO vehicle. Cells were then serum starved for 12 h and stimulated with EGF (50 ng/mL) for 5 min. Cell lysates were prepared, and activated RAS (RAS-GTP) was collected with RAF-RBD-conjugated agarose. The levels of total RAS, H-rev107 fusion proteins, and actin were measured by Western blotting. **(B, D)** HtTA cells were plated in triplicate, incubated overnight, and then transfected for 2 days with control vector, H-rev107 expression vector, and reporter plasmids in the presence of 10 μM AACOCF3, 1 μM palmostatin B, 5 μM MAFP, 2 μM BEL, or DMSO vehicle. Cells were starved and then stimulated with EGF (50 ng/mL) for 12 h at 37°C. Cell lysates were prepared and analyzed for the transactivation activity of ELK1 protein. Representative results from triplicate samples are expressed as mean ± SD. Student’s *t*-test: **, *P* < 0.01; ***, *P* < 0.001.

To discern the role of the downregulation of RAS activation in H-rev107-mediated lipid modification, we determined whether H-rev107-mediated suppression of RAS-GTP was relieved by the PLA/AT inhibitor AACOCF3. EGF increased RAS-GTP levels by 5.4-fold in HtTA cells. The EGF-stimulated RAS-GTP level in H-rev107-expressing HtTA cells was inhibited by 36% compared to control transfected cells (Figure 5A). This suppression was absent in cells treated with AACOCF3. In contrast, AACOCF3 by itself had no effect on the level of RAS-GTP in control cells. Palmostatin B had no effect on the levels of RAS-GTP in either control or H-rev107-expressing HtTA cells (Figure 5A).

We further determined the effect of APT1 or PLA/AT inhibitors on ELK1 transactivation in response to EGF stimulation. In control cells, EGF treatment for 12 h significantly increased ELK1 transactivation. The effect was not altered by co-incubation with AACOCF3 or palmostatin B (Figure 5B). However, EGF-stimulated ELK1 transactivation in H-rev107-expressing cells was decreased by 74%, and the inhibition was alleviated by AACOCF3, but not by palmostatin B (Figure 5B). Therefore, AACOCF3 eliminated H-rev107-mediated suppression of acylated H-RAS. Also, treating cells with AACOCF3 significantly restored H-rev107-mediated suppression of H-RAS-GTP and ELK1 transactivation.

In addition, we also determined the effect of PLA2 inhibitors on H-rev107-mediated H-RAS activation. As shown in Figure 5C and D, treating H-rev107-expressing HtTA cells with AACOCF3 and MAFP increased H-RAS-GTP levels and restored H-RAS-mediated ELK1 transactivation.

## Discussion

The HREV107 protein family has been shown to negatively regulate the activity of RAS [4,7,13,19,20]. Most studies have emphasized the regulation of the downstream signal of RAS by HREV107 protein. Our previous studies indicated that RIG1 or HRASLS2 suppresses RAS-GTP formation [7,13]. Palmitoylation is essential for the trafficking of H-RAS and N-RAS to the plasma membrane. The current study has shown that H-rev107 downregulates H-RAS lipid acylation. This lipid regulation by H-rev107 on RAS may affect subcellular localization and also the RAS activation upon EGF treatment. The results support our previous observation of enhanced Golgi-localized H-RAS in RIG1-transfected cells [13].

Several recent studies demonstrate that each member of the HREV107 protein family may act as a phospholipid-related enzyme to catalyze the release of fatty acid from glycerophospholipid or the transfer of an acyl group from glycerophospholipid to the hydroxyl group of lysophospholipid [6,8,24,25,37]. The acyltransferase activity of HREV107 was mainly focused on N-acylation and O-acylation activities for HRASLS2, H-REV107, and RIG1 [6,37]. Whether the HREV107 protein family can regulate palmitoylation or S-acylation had not been investigated. Our results show that H-rev107 can decrease acylated H-RAS. This observation suggests that H-rev107 possesses S-acyltransferase activity and that H-RAS may be the donor for thioester transfer catalyzed by H-rev107. We hypothesized that H-rev107 can catalyze the formation of deacylated H-RAS from acylated H-RAS. Acyl-H-rev107 intermediate would be then be hydrolyzed to release fatty acid or to transfer the S-acyl group to other cysteine residues of proteins such as Gsα or β2-adrenergic receptor. The acceptors for the S-acyltransferase activity of H-rev107 need to be identified.

H-rev107 activity that suppressed H-RAS acylation is eliminated by AACOCF3 and MAFP, but not by BEL. The inhibitory effect of AACOCF3 and MAFP on H-rev107 is similar to a previous study [3]. The results indicate that PLA/AT activity of H-rev107 is required for both phospholipase activity and the regulation of H-RAS acylation. In contrast, decreasing the deacylated reaction using APT1 inhibitor did not restore the acylated H-RAS level in control or H-rev107-expressing cells. Palmostatin B has been shown to inhibit APT1 activity and perturb the cellular acylation cycle of RAS for a short time [39,44]. The acylation/deacylation cycle of RAS is perturbed by plamostatin B, and this may result in de novo synthesis of RAS and long-term feedback suppression of RAS activity. Furthermore, palmostatin B might suppress RAS activity regardless of its ability to inhibit the deacylation enzyme APT1 [45]. Comparison of the effect of PLA inhibitors and APT1 inhibitor on H-rev107-mediated RAS suppression suggests that the steady-state level of H-RAS within cells, as regulated by H-rev107, can be controlled by PLA, but not by APT1.

Treating cells with AACOCF3 and MAFP resulted in more acylated H-RAS production compared to control cells. Increases in RAS-GTP protein were not observed in cells treated with these inhibitors. Activation of RAS proteins is tightly regulated by GEFs, which exchange GDP for GTP to produce an active, GTP-bound state. GAPs can also catalyze the hydrolysis of bound GTP to turn off RAS [29]. The location for the GDP-GTP cycle for RAS processing is at the plasma membrane, and this subcellular localization is controlled by RAS lipid modification. As shown by the RAS activity assay, AACOCF3 and MAFP may cause more RAS trafficking to the plasma membrane, but these inhibitors had no effect on GEF/GAP activity.

Examination of the enzyme activity of the HREV107 protein family may help to clarify its biological activity. In this study, we demonstrated that the anti-RAS activity of H-rev107 may result from the latter’s PLA/AT activity. Our previous studies also showed that H-rev107 and RIG1 can regulate PGDS activity, which enhances PGD2 production [35,46]. Similarly, PGD2 produced by PGDS has been shown to be required for the activity of phospholipase A [47,48]. Whether H-rev107-mediated regulation of PGDS activity is controlled by the PLA/AT activity of H-rev107 requires further investigation. Posttranslational lipid modification has diverse effects on cellular signaling. Therefore, pleiotropic effects of HREV107 family proteins in the regulation of cellular growth, differentiation, and apoptosis may be intimately associated with their lipid-metabolizing abilities.

There is little literature comparing the effects of each member of the HREV107 family on physiology. H-rev107, HRASLS2, and RIG1, each of which contains a highly conserved NC domain, exert similar biological effects with regard to anti-RAS and proapoptotic activity [5,7,14,20]. Although diverse C-terminal transmembrane domains are present in each member of the HREV107 family, all family members localize to similar organelles within cells, such as the endoplasmic reticulum or Golgi apparatus [7,9,19]. Nazarenko et al. showed that H-rev107 in the nucleus stimulates the growth of non-small cell lung carcinoma [49]. Similarly, proapoptotic activity was observed with cells that expressed full-length or truncated RIG1 that appeared in the perinuclear region or nucleus, respectively [14]. In addition to cellular function, tissue distribution should also be considered. H-rev107 and RIG1 were shown to be expressed similarly except in peripheral leukocytes, whereas HRASLS2 is expressed only in liver, kidney, testis, intestine, and colon [6]. The different expression profiles of the HREV107 family in humans might reflect tissue-specific activity or the redundancy of gene evolution.

The relation of the phospholipid-metabolizing activity of the HREV107 protein family to its anti-tumor activity remains unclear. Here we provide insight into the phospholipid-metabolizing activity of H-rev107, which regulates RAS palmitoylation and thereby affects RAS activity. Metabolites produced by HREV107 protein and/or the enzyme activity itself are likely to play roles in inducing cellular differentiation and suppressing cellular growth. Analysis of PLA/AT activity of the HREV107 family proteins in other known processes, such as proapoptosis [5,7,14] or transglutaminase [15,22] or in target molecules such as PGDS [35,46] would help elucidate the mechanism responsible for the biological functions of the H-REV107 family proteins.

## Conclusions

Our study revealed that H-rev107 exerts its anti-RAS activity through its PLA/AT activity to decrease the steady state levels of H-RAS palmitoylation. Suppression of PLA/AT activity alleviates the H-rev107-mediated suppression of RAS activity, which to our knowledge has not been investigated previously.

## Abbreviations

AACOCF3: Arachidonyl trifluoromethyl ketone; APT1: Acyl-protein thioesterases 1; BEL: Bromoenol lactone; EGF: Epidermal growth factor; GAPs: GTPase-activating proteins; GEFs: Guanidine nucleotide exchange factors; MAFP: Methyl arachidonyl fluorophosphate; PLA/AT: Phospholipase A/acylatransferase; RIG1: Retinoid-inducible gene 1.

## Competing interests

The authors declare that they have no competing interests.

## Authors’ contributions

C-HW designed research and performed and drafted the experiments; C-CW, R-YS, L-KW, T-CT, and M-LC designed the research and data discussion; S-YJ supervised the experiments and assisted in the writing of and proofed the manuscript; F-MT performed the experiments, contributed to experimental design, and drafted the manuscript. All authors read and approved the final draft of the manuscript.
